# Spontaneous Retroperitoneal Bleeding in a Patient with Primary Antiphospholipid Syndrome on Aspirin

**DOI:** 10.1155/2018/4397893

**Published:** 2018-09-06

**Authors:** Petros Ioannou, George Alexakis

**Affiliations:** ^1^Internal Medicine Department, University Hospital of Heraklion, Crete, Greece; ^2^Emergency Department, University Hospital of Heraklion, Crete, Greece

## Abstract

Retroperitoneal bleeding is a rare and difficult to diagnose condition, defined as bleeding in the retroperitoneal space without associated trauma or iatrogenic manipulation. It has been associated with hematologic diseases and malignancies and is more common in patients receiving systemic anticoagulation. A 50-year-old man on aspirin presented with abdominal pain. Physical examination revealed abdominal tenderness and a palpable mass at the left abdominal area. An abdominal CT revealed a spontaneous retroperitoneal hematoma due to bleeding of an intraparenchymal branch of the left renal artery. The patient underwent left nephrectomy in order to control the bleeding. Pathology of the kidney showed evidence of acute and chronic microangiopathy, renal artery stenosis, and renal vein thrombosis. Further work-up led to diagnosis of primary antiphospholipid syndrome. Treatment of spontaneous retroperitoneal bleeding varies from conservative in hemodynamically stable patients to invasive or even surgery in hemodynamically unstable patients. In our case, open surgery was performed due to the rapidly deteriorating patient's condition and the inability to embolize the bleeding vessel by interventional radiology. Physicians should always think of retroperitoneal bleeding in patients presenting with abdominal pain and signs of hypovolemia, especially if they have a bleeding disorder or receive anticoagulants or antiplatelets.

## 1. Introduction

Retroperitoneal bleeding is a well-recognized but rare and difficult to diagnose condition, defined as bleeding in the retroperitoneal space without associated trauma or iatrogenic manipulation. It has been associated with hematologic diseases, malignancies, renal angiomyolipomas or infarction, and Evans syndrome [1–3], while it has been shown to be more common in patients receiving systemic anticoagulation, as shown in 2 recent studies [4, 5]. Compared to other sites of bleeding, retroperitoneal bleeding can be very difficult to suspect, due to the minimal specific symptoms that are associated with this condition, while its diagnosis and treatment also pose difficulties. Thus, its diagnosis and management are often triggered by the hemodynamic compromise in a patient with no other obvious site of bleeding. Herein, we report the case of a young patient who presented with spontaneous retroperitoneal bleeding due to spontaneous renal hemorrhage.

## 2. Case Presentation

**Figure 1:**
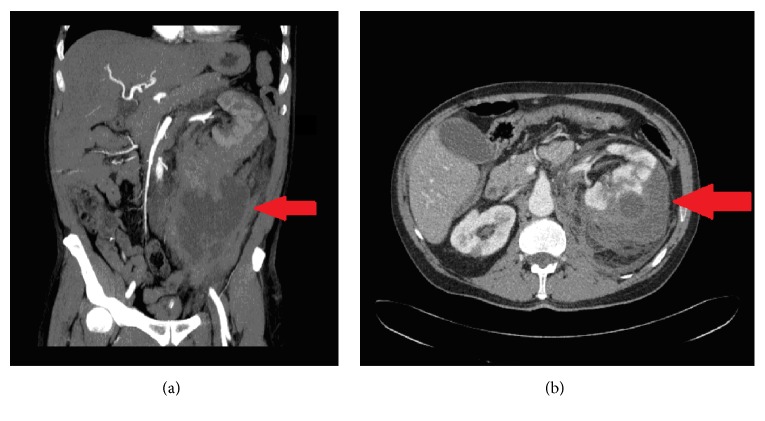
A coronary and a horizontal view of an abdominal CT with intravenous contrast that revealed a 17x11x8cm^3^ spontaneous retroperitoneal hematoma (shown with arrow) due to bleeding of an intraparenchymal branch of the left renal artery extending from the left kidney down to the pelvis are shown ((a) and (b), respectively).

A 50-year-old Caucasian man with a remote history of deep venous thrombosis (DVT) at the lower extremities presented with abdominal pain. His medications included acetylsalicylic acid. Physical examination revealed abdominal tenderness and a palpable mass at the left abdominal area. Laboratory exams revealed a normocytic and normochromic anemia, with a hemoglobin of 11.1g/dl (reference range 14–18 g/dl), aPTT and INR prolongation of 78.1 seconds and 1.64, respectively (reference range 25-36 seconds and 0.85 – 1.2, respectively), and elevated urea and serum creatinine values of 68 mg/dL and 1.8mg/dL, respectively (reference range 15–55 mg/dL and 0.7–1.3mg/dL, respectively). An abdominal computerized tomography (CT) revealed a 17x11x8cm^3^ spontaneous retroperitoneal hematoma due to bleeding of an intraparenchymal branch of the left renal artery (Figure 1). A repeat hemoglobin after 2 hours was 9.1g/dL and the patient was transfused with 2 units of packed red blood cells and 2 units of fresh frozen plasma, while a central venous catheter, a urinary catheter, and an arterial catheter were placed to allow for hemodynamic monitoring. Left nephrectomy was performed due to inability to embolize the bleeding artery. Pathology of the kidney showed evidence of acute and chronic microangiopathy, renal artery stenosis, and renal vein thrombosis. Antiphospholipid antibodies and lupus anticoagulant were positive twice, while antinuclear antibodies and anti-ds-DNA were negative, suggesting the diagnosis of primary antiphospholipid syndrome.

## 3. Discussion

Retroperitoneal bleeding is a medical emergency that is often difficult to diagnose due to its rarity and the nonspecific symptoms with which it presents. It can be caused by several causes, such as trauma, malignancy, iatrogenic manipulation, ruptured abdominal aneurysm, and coagulopathy; however spontaneous retroperitoneal bleeding is a unique entity with few cases reported in the literature [4]. Most published cases involve patients receiving anticoagulants, while some of them were receiving antiplatelets at the same time, while only a small minority of the patients with spontaneous retroperitoneal bleeding was receiving only antiplatelet drugs [4–6]. The pathophysiological mechanism of spontaneous retroperitoneal bleeding is not quite clear; however, there are studies suggesting that diffuse occult vasculopathy and atherosclerosis of the small vessels in the retroperitoneal space could lead to rupture of the most friable vessels among them [7]. On the other hand, some suggest that even though the name “spontaneous” implies no trauma, it could be that minor unrecognized injury, such as in intense coughing or vomiting, or minor trauma in sports could lead to a forceful muscular strain that may progress to retroperitoneal bleeding [7, 8]. In our case, biopsies from the kidney after nephrectomy revealed the presence of microscopic and macroscopic vascular changes which could have led to the development of spontaneous bleeding, especially in this patient who could have been predisposed to bleeding spontaneously due to antiplatelet therapy.

Treatment of spontaneous retroperitoneal bleeding may vary. To our knowledge, there are no specific guidelines to reliably suggest when a conservative approach may not be enough, and endovascular or surgical management should be attempted. For example, in the case of spontaneous retroperitoneal bleeding in a hemodynamically stable patient who was on anticoagulants, a conservative approach with correction of the coagulation abnormalities, supportive measures, and volume resuscitation could suffice for the management of this condition [7]. Interventional treatment in the case of retroperitoneal bleeding involves intra-arterial embolization in case active bleeding is recognized on angiography [9–11], and open surgery is usually reserved for the cases where interventional radiology is unsuccessful or unavailable or if the patient develops abdominal compartment syndrome [7, 12]. In our case, open surgery was performed due to the rapidly deteriorating patient's condition and the inability to embolize the bleeding vessel by interventional radiology.

Collectively, we describe the case of a patient who was on chronic treatment with aspirin, spontaneously developed retroperitoneal bleeding, and was treated with nephrectomy in order to control bleeding. Physicians should always think of retroperitoneal bleeding in patients presenting with abdominal pain and signs of hypovolemia, especially if they have a bleeding disorder or receive anticoagulants or antiplatelets.
